# A novel proinflammatory role for granzyme A

**DOI:** 10.1038/cddis.2017.56

**Published:** 2017-02-23

**Authors:** Jacqueline A van Eck, Liling Shan, Jan Meeldijk, C Erik Hack, Niels Bovenschen

**Affiliations:** 1 Department of Pathology, University Medical Center Utrecht, Utrecht 3584 CX, The Netherlands; 2 Laboratory of Translational Immunology, University Medical Center Utrecht, Utrecht 3584 CX, The Netherlands

Granzymes are a family of homologous serine proteases involved in inducing apoptosis in virus-infected cells and tumor cells.^1, 2^ In humans, five granzymes (GrA, GrB, GrH, GrK, and GrM) are expressed, and stored in granules of natural killer (NK) cells, NK-T cells, cytotoxic T cells, and *γδ* T cells, which are collectively referred to as cytotoxic lymphocytes.^1, 2^ After release of the granule content in the immunological synapse between a target cell and a cytotoxic lymphocyte, granzymes enter the cytosol of the target cell with the aid of pore-forming protein perforin. Inside the target cell, granzymes cleave various death substrates.^1, 2^ Macrophages, mast cells, and dendritic cells can also express granzymes but not perforin, suggesting perforin-independent (extracellular) roles of granzymes.^3^

Cumulative evidence is emerging that (extracellular) granzymes modulate inflammation.^3^ Patients suffering from inflammatory diseases, such as rheumatoid arthritis, sepsis, and viral or bacterial infections, have elevated levels of granzymes in their synovial fluid, plasma, serum, and/or broncheoalveolar lavage fluid.^3, 4^ It has been recently demonstrated that GrM is involved in the early stages of mucosal inflammation, since GrM-knockout mice display enhanced inflammation in a mouse model of ulcerative colitis.^5^ Extracellular GrA and GrK can directly release proinflammatory cytokines from monocytes, macrophages, and fibroblasts (Figure 1).^6, 7, 8, 9, 10^ These effects are dependent on the granzyme catalytic activities and are at least partly enhanced upon granzyme intracellular delivery.^6, 7, 8, 9, 10^ The molecular mechanisms by which granzymes directly release proinflammatory cytokines largely remain unclear. While GrK can cleave and activate the protease-activating receptor 1 (PAR1) leading to cytokine release from fibroblasts,^10^ GrA can convert pro-IL-1*β* into bioactive IL-1*β* (Figure 1).^11, 12^ In human monocytes, this latter process depends on caspase-1 activity (inflammasome), but in human blood-derived macrophages, the caspase-1 pathway appears not to be involved.^11^

Granzymes can also interplay with LPS-TLR4-induced cytokine response during the antimicrobial innate immune response.^3^ Evidence for this comes from observations that mouse GrA^8^ and mouse GrK^7^ induce IL-1*β* release in macrophages that have been sensitized with LPS. Furthermore, GrA- and GrM-knockout mice survive longer than WT mice when challenged with LPS,^6, 8, 13^ and GrM-knockout mice produce less cytokine upon LPS injection.^13^ Finally, GrK can synergistically enhance LPS-induced cytokine release *in vitro* from primary human monocytes and *in vivo* in a mouse model of LPS challenge.^9^ Intriguingly, these extracellular effects are independent of GrK catalytic activity. GrK binds to LPS, disaggregates LPS from micelles, and augments LPS–CD14 complex formation, thereby likely boosting monocyte activation by LPS.^9^ Whether other granzymes than GrK can also directly augment TLR4 signaling during the antimicrobial innate immune response remains unknown.

In our recent paper published in *Cell Death Discovery*,^14^ we have examined the role of GrA in potentiating TLR signaling and cytokine release from human monocytes. Extracellular GrA alone showed minor, if any, cytokine response from monocytes, but treatment of monocytes with GrA in combination with TLR2- and TLR4-agonists caused a marked increased release of proinflammatory cytokines TNF*α*, IL-6, and IL-8. GrA also potentiated the release of TNF*α* from monocytes incubated with Gram-negative bacteria. Interestingly, a catalytically inactive mutant of GrA resulted in similar cytokine release as compared with WT GrA, indicating that this process is not dependent on GrA catalytic activity (Figure 1). To determine whether CD14-dependent signaling is involved, monocytes were pre-incubated with a neutralizing CD14 antibody before treatment with GrA and LPS. This resulted in the absence of a cytokine response, indicating that GrA-enhanced cytokine release depends on CD14 signaling. Unlike GrK, GrA did not bind to LPS, did only marginally liberate LPS molecules from micelles, and did not stimulate LPS–CD14 complex formation. These results indicate that granzymes can use different mechanisms to enhance LPS-induced cytokine release from monocytes.^9, 14^

Apparently, GrA potentiates TLR-mediated cytokine response independent of its catalytic activity, while GrA can also use its proteolytic activity to release cytokines in the absence of TLR stimulation (Figure 1). It has been well established that granzyme activity *in vivo* is tightly regulated by serine protease inhibitors (serpins) and that extracellular GrA in complex with proteoglycans is resistant for inactivation by serpins.^15^ This raises the possibility that regulation of granzyme activity is essential to fine-tune the proinflammatory cytokine response.

The molecular mechanism by which GrA – irrespective of its catalytic activity – potentiates TLR-agonist-induced proinflammatory cytokine release remains unknown. GrA may act extracellularly, for example, via binding to (cell surface) molecules to boost TLR signaling and/or GrA may be taken up by monocytes to fulfill an intracellular function in stimulating (canonical) TLR signaling (Figure 1). Since GrA did not affect LPS-induced IFN*β* release, it seems conceivable that GrA enhances cytokine release by affecting the MyD88 pathway. Further study is required to discriminate between these possibilities.

Our recent paper in *Cell Death Discovery*^14^ further strengthens the contention that granzymes can modulate TLR signaling and proinflammatory cytokine response during bacterial infection. This functional redundancy may ensure the development of a proper antibacterial innate immune reaction. Therapeutic intervention of granzyme-induced cytokine release might in the future contribute to treatment of inflammatory diseases.

## Figures and Tables

**Figure 1:**
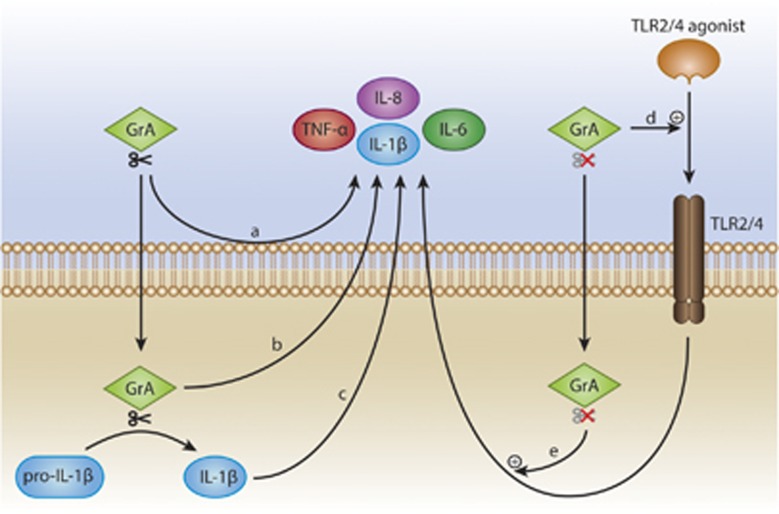
Model of GrA functions in cytokine release and TLR signaling. (a) Extracellular GrA can directly release proinflammatory cytokines, dependent on its catalytic activity.^6, 7, 8, 9, 10^ (b) This cytokine release is at least partly enhanced upon granzyme intracellular delivery.^7, 8^ (c) In addition, intracellular GrA can convert pro-IL-1*β* into bioactive IL-1*β*, which is secreted.^11, 12^ Furthermore, GrA potentiates TLR2/4 agonist-induced proinflammatory cytokines independent of its catalytic activity.^14^ (d) It may act extracellularly, for example, via binding to (cell surface) molecules to boost TLR signaling and/or (e) GrA may be taken up to fulfill an intracellular function in stimulating TLR signaling. (GrA with scissors: catalytic activity of GrA is required; GrA with scissors crossed out: catalytic activity of GrA is not required.)
